# Contribution of the slow motion mechanism to global motion revealed by an MAE technique

**DOI:** 10.1038/s41598-021-82900-2

**Published:** 2021-02-17

**Authors:** Satoshi Shioiri, Kazumichi Matsumiya, Chia-huei Tseng

**Affiliations:** 1 Research Institute of Electrical Communication, Tohoku University, 2-1-1 Katahira, Aoba-ku, Sendai, 980-8577 Japan; 2 Department of Applied Information Sciences, Tohoku University, Sendai, Japan; 3 Graduate School of Information Sciences, Tohoku University, Sendai, Japan

**Keywords:** Sensory processing, Motion detection

## Abstract

Two different motion mechanisms have been identified with motion aftereffect (MAE). (1) A slow motion mechanism, accessed by a static MAE, is sensitive to high-spatial and low-temporal frequency; (2) a fast motion mechanism, accessed by a flicker MAE, is sensitive to low-spatial and high-temporal frequency. We examined their respective responses to global motion after adapting to a global motion pattern constructed of multiple compound Gabor patches arranged circularly. Each compound Gabor patch contained two gratings at different spatial frequencies (0.53 and 2.13 cpd) drifting in opposite directions. The participants reported the direction and duration of the MAE for a variety of global motion patterns. We discovered that static MAE durations depended on the global motion patterns, e.g., longer MAE duration to patches arranged to see rotation than to random motion (Exp 1), and increase with global motion strength (patch number in Exp 2). In contrast, flicker MAEs durations are similar across different patterns and adaptation strength. Further, the global integration occurred at the adaptation stage, rather than at the test stage (Exp 3). These results suggest that slow motion mechanism, assessed by static MAE, integrate motion signals over space while fast motion mechanisms do not, at least under the conditions used.

## Introduction

The analysis of motion signals is one of the most important functions of early vision^1,2^. The motion information is used in a variety of visual processes such as the segregation of moving objects from the background, the recovery of 3D shapes from velocity fields caused by object motion, and the visual perception of self-motion provided by expansion/contraction motion on the retina. These processes integrate motion signals detected locally to compare the velocities at different locations. Different kinds of local motion integration have been considered, and a range of motion analyses have been identified, e.g., relative motion^3–5^, translation, rotation, expansion/contraction^6–8^, motion parallax^9,10^, shape from motion^11,12^, and motion in depth induced by interocular velocity differences^13–20^.

Multiple motion sensitive mechanisms (local motion detectors) with different spatial frequency tunings in early visual processes have been identified to account for local motion detection^21^. In the temporal domain, two types of local motion detectors are often assumed that are distinct in terms of temporal characteristics^22–29^. Among these studies, Shioiri and Matsumiya^23^ developed a motion aftereffect (MAE) technique to isolate fast and slow motion mechanisms with a test stimulus under different temporal conditions. Their results revealed several characteristic differences between the two motion mechanisms: temporal frequency tuning, spatial frequency tuning, orientation tuning and sensitivity to relative motion. Fast motion mechanisms are more sensitive to higher temporal frequency and lower spatial frequency. Slow motion mechanisms are more sensitive to relative motion and have narrower orientation tuning than fast motion mechanisms. To process, for example, rotation, a variety of direction signals should be integrated and relative motion process is a part of such integration. Sensitivity to relative motion is effective to spatially integrate motion direction signals, and narrow orientation tuning provides more precise direction information coding. These properties enable slow motion mechanisms to contribute more to global motion with complex spatial variations, such as rotation, expansion/contraction, biological motion than fast motion mechanisms.

There are reports that suggest that both the fast and slow motion mechanisms contribute to global motion perception^24,30^. Edwards et al.^30^ used the detection of coherently-moving (signal) dots among randomly moving (noise) dots at different speeds to reveal participants’ motion integration over a large space. Fast-moving noise dots impaired only fast-moving signal dots and slow-moving noise dots impaired only slow-moving signal dots. This selective masking effect indicates that both the slow and fast motion mechanisms contribute to global motion. van der Smagt, Verstraten, and van de Grind^24^ reported a transparent motion aftereffect (MAE) caused by the simultaneous adaptation of dynamic random dots moving at slow and fast velocities. The co-existence of the MAEs of two adapting motion components implies independent motion mechanisms coding of global motion. These studies suggest that both fast and slow motion signals can be used for processing global motion.

There are different functions in global motion analysis including self-motion analysis, structure identification, and depth from motion. The appropriate speed for analyses depends on the task requirements because the optimal speed may differ for different functions. A large field filled with high speed stimuli, which is typically used in global motion studies, may be appropriate for a self-motion analysis as we incorporate a whole-range optic flow to compute our body movements. In contrast, smaller, slow speed stimuli with details may be appropriate for a structure for motion analysis as it does not require self-position and movement calculations. In the present study, we focus on a less studied condition with a relatively small and detailed spatial information and examine the relative contributions made by fast and slow motion mechanisms to global motion. We used a MAE to isolate the slow and fast motion mechanisms. The MAE is a powerful technique with which to isolate one motion mechanism from the others as shown in the studies that isolated global motion from local motion^6,7^. The present MAE paradigm was built on the technique developed by Shioiri and Matsumiya^23^. The adapting stimulus comprises two superimposed sinusoidal gratings with different spatial frequencies (2.1 and 0.53 c/deg), moving in opposite directions. After exposure to the moving stimulus, the MAE is measured with separate static and flicker tests.

In this study, we define the motion mechanism sensitive to static MAE as the slow motion mechanism and that sensitive to the dynamic MAE as the fast motion mechanism. They may also be called as high spatial frequency motion mechanism and low spatial frequency motion mechanism if we consider the results in Shioiri and Matsumiya^23^. It was shown that the static MAE moved in a direction opposite to that of the high spatial frequency grating, and the dynamic MAE moved in a direction opposite to the low spatial frequency grating. It is important to note that the same phenomenon was found under the condition where the adapting high spatial frequency motion is faster in speed (i.e. 9.4 deg/s) than low spatial frequency motion (i.e. 2.4 deg/s, see Fig. 10 in Ref. ^23^). This appears to contradict the interpretation of different MAEs between static and flicker test by slow and fast motion mechanisms. However, MAE occurs whenever adaptation and test stimuli are processed by a shared mechanism. Our definition of slow/fast motion here is not based on adaptation stimuli speeds, but on temporal properties of test stimuli (static vs flicker stimuli), derived from the temporal frequency tuning measured for the two systems^23^. The motion mechanism sensitive to static test respond to very slow motion while the motion mechanism sensitive to flicker test does not (Fig. 8 of Ref. ^23^).

**Figure 1:**
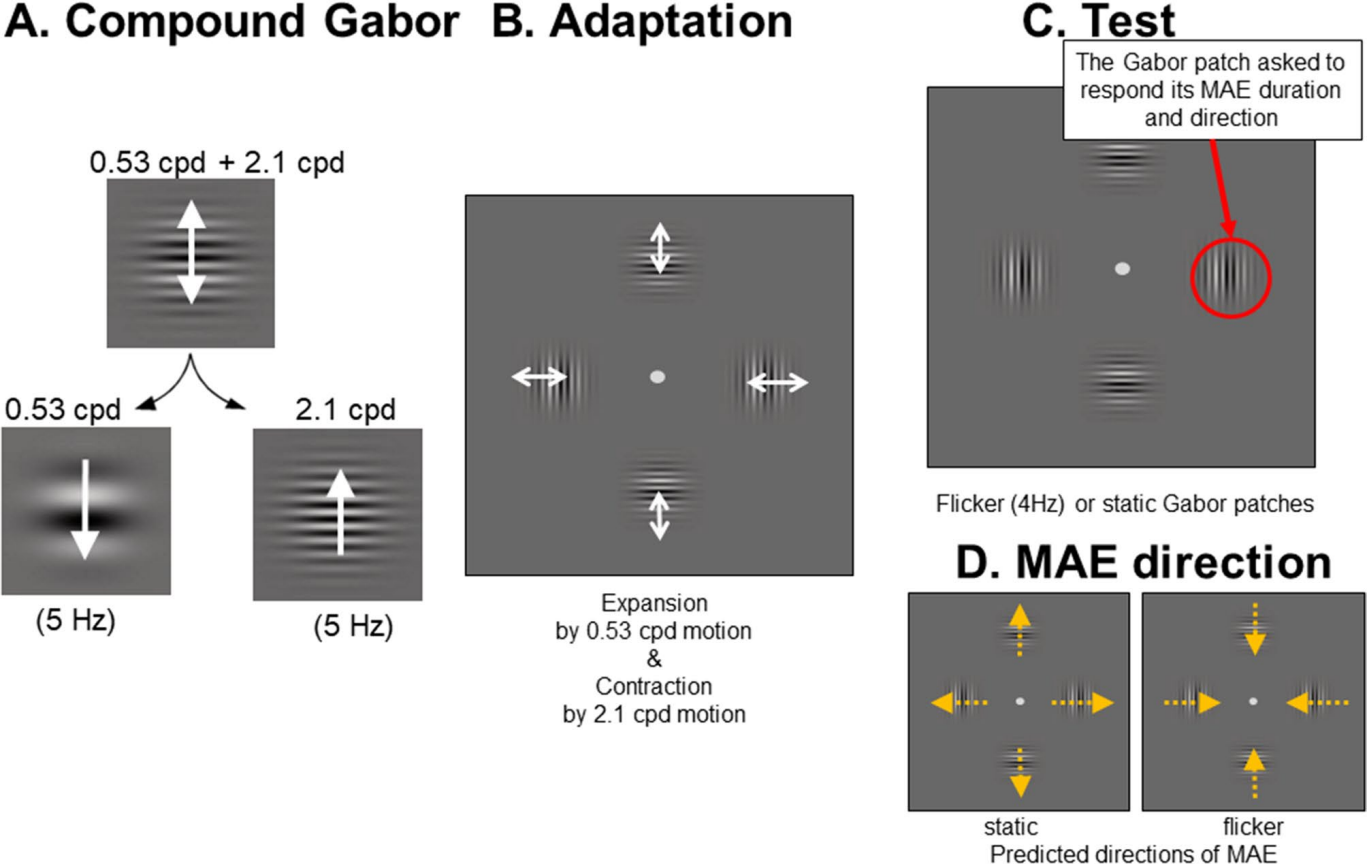
Stimuli used. (**A**) Each compound motion element was superimposed with two sinusoidal gratings: one at a spatial frequency of 0.53 cpd and the other at 2.13 cpd. The two gratings drifted within the Gabor patch in opposite directions at 5 Hz. For the superimposed Gabor patch, participants reported perceiving the motion direction of the 0.53 cpd grating. (**B**) Four component Gabor patches at fixed locations (above, below, to the left, and to the right of the central fixation) were presented during the adaptation period. The arrow in the example showed the motion directions of the 0.53 cpd grating. When the motion elements were integrated across all locations, the participants perceived global expansion. (**C**) During the test period, four Gabor patches at the same locations in the adaptation period either flickered at 4 Hz or were static. The participants responded to both the perceived direction and the duration of the perceived motion. (**D**) MAE direction can be predicted as shown from the previous study. (Note: the color of the grating was modulated along yellow with a yellow background).

We arranged compound Gabor patches in space so that their spatial configurations generated global motion (Fig. 1). The perceived directions and durations of MAEs were measured in three experiments to investigate the effect of global motion on slow and fast motion mechanisms separately. MAE is a good method to investigate temporal characteristics of multiple motion mechanisms in isolation both in psychophysical^31^ and electrophysiological experiments^32^, including the slow and fast motion mechanisms here, referring different temporal frequency tunings^23,33^.

## General method

In the first experiment, we generated 7 kinds of global motion to identify the best types of global arrangements for MAE observations. In the second experiment, we modulated the strength of the global motion during adaptation. In the third experiment, we examined at which stage the global motion integration occurred: during adaptation, or during the test. The static and flicker MAE durations were measured in all experiments.

If the motion mechanism underlying the MAE is sensitive to global motion perception, we expect a longer MAE with a stronger global motion adaptation, whereas we expect no such change in MAE duration if the underlying mechanism is sensitive only to local motion. If the slow motion mechanism contributes to global motion but the fast motion mechanism does not, the global motion strength should modulate the static MAE duration while no such effect is expected for flicker MAE.

To investigate the effect of the slow and fast motion mechanisms on global motion separately, we arranged compound Gabor motion elements in a circle to provide global motion signals (Fig. 1). Each compound motion element (Fig. 1A) consisted of two superimposed Gabor patches differing in carrier spatial frequency (high and low spatial frequencies) and in motion direction (always in opposite to each other). Each Gabor patch was composed of two sinusoidal gratings drifting in opposite directions at 5 Hz. The spatial frequencies of the sinusoidal gratings were 0.53 cpd and 2.1 cpd. The patch size was determined by the space constant (σ) of the Gaussian envelope of the Gabor which was set to 1 deg. Each patch was drawn within the area of ± 2 σ horizontally and vertically. The contrast of each grating was adjusted so that it was 30 times higher than the detection threshold measured with the method of adjustments. The detection threshold measurement for each type of stimuli was conducted in Exp. 1, In Exp. 2 and 3, the detection threshold for four patch stimuli were used for all stimulus conditions. A contrast threshold, defined as the minimum contrast needed for the subjects to see the motion in the Gabor, was measured separately for high and low spatial frequency stimuli drifting at 5 Hz and then used in the main experiments to equate the relative contributions of high and low spatial frequency gratings to motion. Threshold contrasts for the two directions were averaged. A global motion percept from the integration of these local elements was generated (Fig. 1B, supplementary video file 1). After prolonged fixation, a global motion aftereffect was generated, which will be used to estimate the relative contribution from slow and fast motion mechanisms from the difference between conditions with and without global motion in adaptation stimuli.

## Experiment 1: global motion types

### Participants

Five male volunteers were recruited as participants. All the participants had normal or corrected-to-normal vision and, except for one who was an author of this work, were kept naïve about the purpose of the experiment. Informed consent was obtained from each participant when they understood the procedures of this study. This study was approved by the Ethics Committee of the Research Institute of Electrical Communication, Tohoku University, and the experiment was carried out in accordance with the Code of Ethics of the World Medical Association (Declaration of Helsinki).

### Apparatus

The stimuli were presented on a display (G520, Sony) (1024 × 768 pixels and 80 Hz non-interlaced) controlled by a video card (ViDaGe, Cambridge Research) and a computer. The participants were 38 cm from the display, making each pixel subtend 3.4 arcmin. The participant’s head was held steady with a chin rest.

### Stimulus and procedure

**Figure 2:**
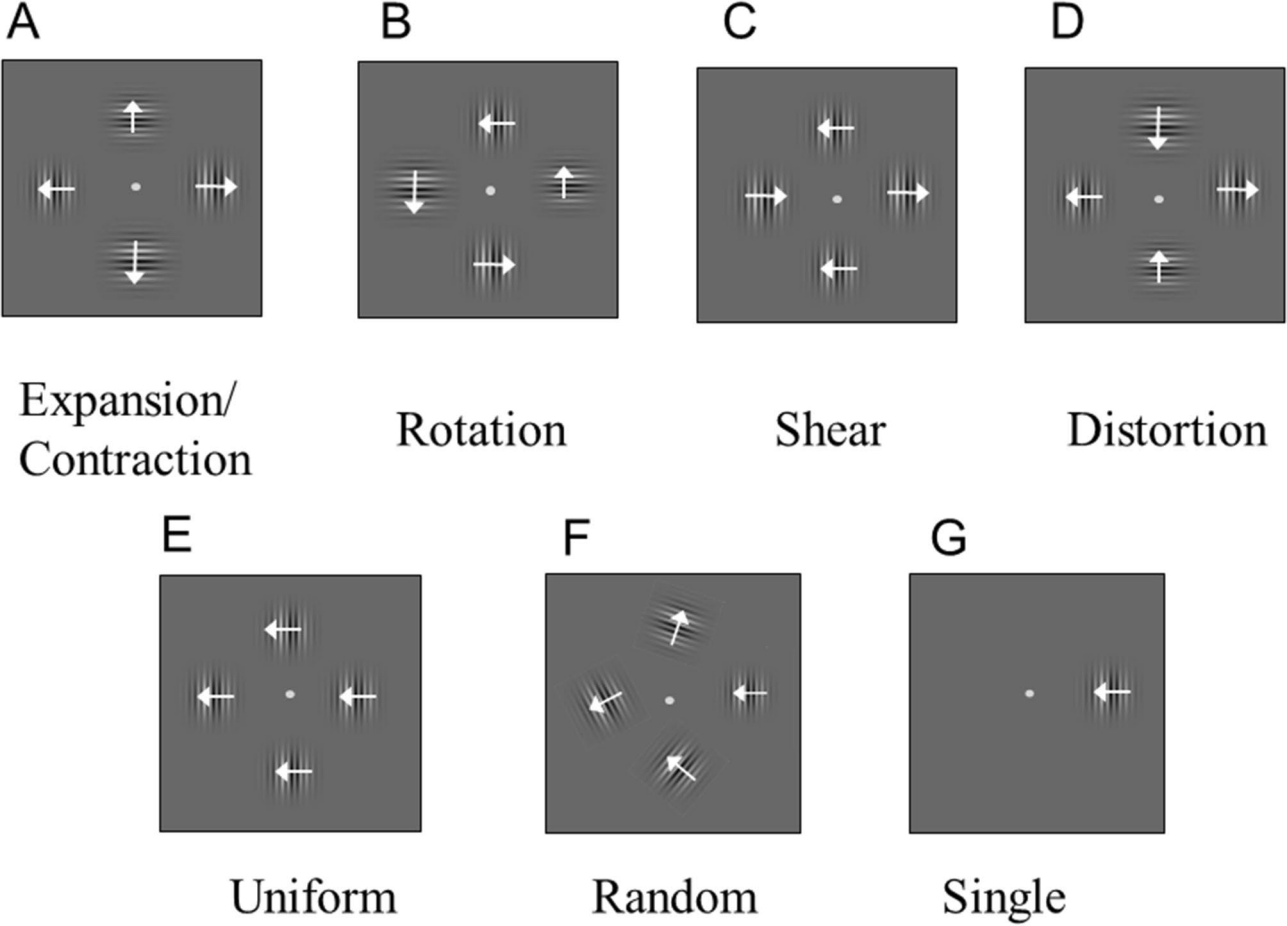
Adaptation stimuli in Experiment 1. The arrows showed the motion directions of the 0.53 cpd grating and the components of 2.13 cpd drifted in opposite directions or vise versa. (**A**) Expansion/Contraction Condition: four identical compound motion Gabor patches drifted away from the center (thus creating an expansion, as shown by the arrows) or toward the center (thus creating a contraction motion). (**B**) Rotation Condition: four compound motion Gabor patches with different drifting directions in a circular arrangement when a global rotational motion (counter-clockwise as shown by the arrow) was perceived. (**C**) Shear Condition: The top and bottom Gabor patch pair moved in opposite directions to the left and right Gabor patch pair. (**D**) Distortion Condition: The two vertical Gabor patches move toward the center while the two horizontal Gabor patches move away from the center. (**E**) Uniform Motion Condition: all four Gabor patches move in the same direction. (**F**) Random Motion Condition: the four Gabor patches drift in no systematically defined directions except that one patch moved horizontally so that the MAE direction was either left or right. The other three moved in directions randomly selected from angles between 30 and 330 degrees with a separation of 30 degrees from trial to trial. We regenerate the stimulus in the cases that the arrangement resembled any of the other types of arrangement. (**G**) Single element condition: A single Gabor patch was presented at the right of the fixation and it moved horizontally.

Participants watched an adaptation display for 20 s before the test display was presented. In the adaptation display, four Gabor patches were placed above, below, to the left, and to the right of the fixation point. The same contrasts were used in both the adaptation and test phases. The adaptation stimuli (i.e. 4 Gabor patches) were circularly arranged at 4 degrees from the center (center to center distance) to provide the sensation of different types of global motion such as rotation, expansion/contraction, shear, uniform, and distortion (Fig. 2A–E). When the motion direction was away/toward from the center, the perceived global motion direction was expansion/contraction (Fig. 2A, Expansion/Contraction Condition). When the motion direction of an element differed by 90 deg from its neighbors’ motion direction, the perceived global motion direction was rotational (Fig. 2B, Rotation Condition). When the motion direction differed by 180 deg from its neighbors’ motion direction, the motion of the middle row was perceived as being opposite to that of the top and bottom rows (Fig. 2C, Shear). When the middle column of elements moved toward the center, and the side columns moved away from the center, the perceived global shape was a distorted square (Fig. 2D, Distortion Condition). When the four elements moved in the same direction this was termed Uniform Condition (Fig. 2E). We also included random motion and a single Gabor patch condition as a baseline control. In the random motion condition, we used the configuration shown in Fig. 2F, where the right patch always moved horizontally so that the MAE direction was either left or right while the other three Gabor patches drifted in no systematically defined directions randomly selected, from trial to trial, from angles between 30 and 330 degrees with a separation of 30 degrees. We regenerate the stimulus in the cases that the arrangement resembled any of the other types of arrangement. In the Single element condition (Fig. 2G), we presented a single compound Gabor patch to the right of the fixation and it moved horizontally.

Although two possible global motion percepts were generated by low-spatial frequency components and high-spatial frequency components respectively, the dominant global motion perceived by the participants was that of the 0.53 cpd grating. Occasionally, the participants reported transparent motion. Both the Gabor and background stimulus colors were yellow with CIE xy color coordinates of (0.413, 0.506), and with a luminance modulation to shape the Gabor function. We used yellow because our plan was to extend the study to equiluminant color gratings in future experiments. We followed the procedure mentioned in general method to determine the grating contrast. The actual contrasts used was 37.1% on average for all types of motion stimuli of all participants, and the standard deviation of motion types averaged over all participants was 11.4% for the high SF grating motion. For the low SF grating motion, they were 25.5% and 6.2%. The background size was 17.3 × 30 degrees, which was the size of the display used.

The test display started with the last frame of the adaptation stimulus and contained 4 identical compound Gabor patches as the adaptation display, with the phase when the adaptation motion stopped. The test temporal condition was either static (no change until the response) or flickering at 4 Hz (sinusoidal counter phase flickering until response). Participants responded at the moment when the MAE disappeared (i.e. duration). They were instructed to indicate the MAE direction as well using two keys, based on the Gabor at 3′clock location because this is the location for the only Gabor adaptor in the single element condition. This allowed us to use a criterion independently of stimulus types or of patch numbers. What we expected here was that the participant would report MAE of a single patch when they see global MAE as well as they see MAE of the patch locally. While our results do not separate the two, we can identify the effect of global motion either directly seeing global motion in MAE or indirect effect from global motion analysis on the single patch MAE. No difference was expected if there were no effect of global motion. Although asking MAE of global motion was another possibility, criterion for global motion could be difficult due to variation of MAE of different patches.

The adaptor (and thus the MAE) direction was either left or right except for rotation where it was either up or down. There was a third key that indicated no MAE perceived, and the response was regarded as being an MAE duration of 0 s. Each participant repeated the experiment 12 times under each of the seven motion conditions. Different motion types were used in different blocks in a randomized order. We expected the longer MAE if there would be global motion influences on the MAE and the effect was compared among different stimulus conditions directly since we used an identical task to report the MAE of the Gabor at 3′clock location for all stimulus conditions.

### Results

**Figure 3:**
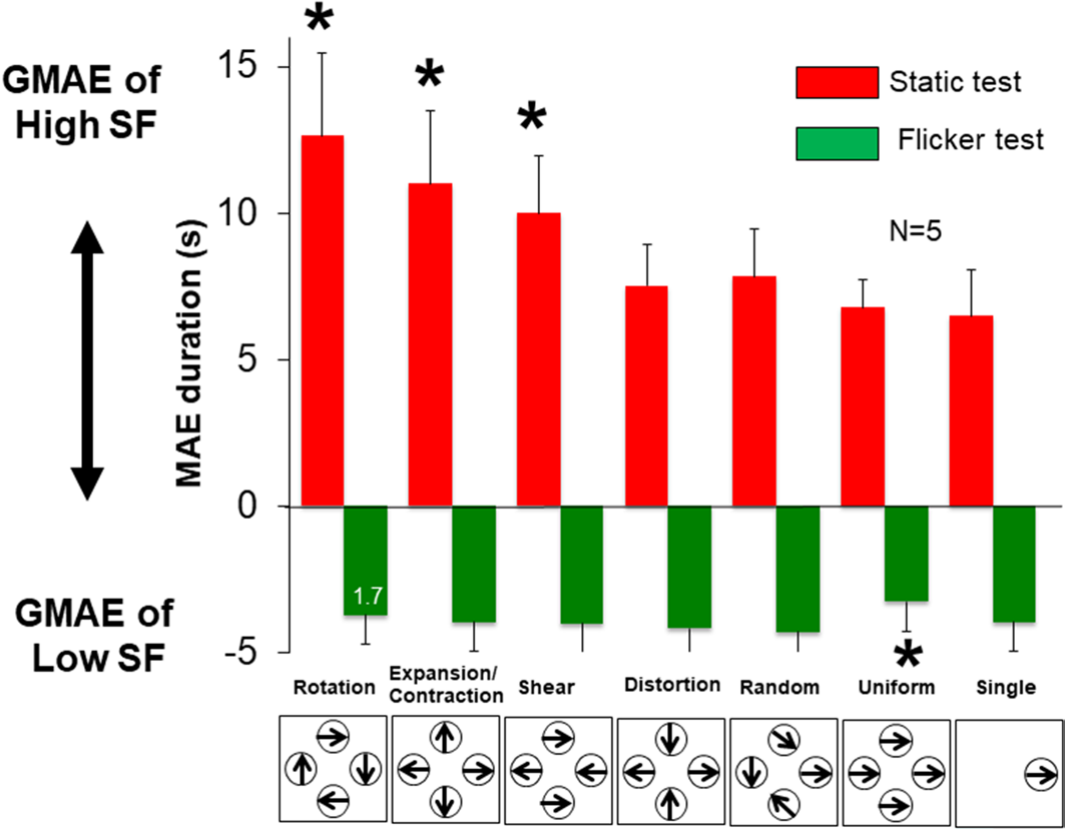
Results for Experiment 1. The MAE duration is plotted for different motion conditions. The MAE from the integration of high spatial frequency components is labeled as positive, and negative values indicate the MAE direction induced by the integration of low spatial frequency components. Asterisks indicate that the MAE duration is statistically significantly different from that of the single condition. In conditions where there were responses opposite to the expected MAE direction, the percentage of time that those occurred is given as a number on the data bar for that condition. This only occurred with the flicker test for the Rotation condition in this experiment.

The results are summarized in Fig. 3 (MAE duration in second) and Table 1 (Z-score and Hedges' g as effect size of difference from the Single condition). MAE directions responded were consistent with the prediction at almost all trials. When there were the responses in opposite direction, the percentage of responses in unpredicted direction was provided in Fig. 3 (e.g. 1.7% at Rotation with Flicker Test, Fig. 3) and was included in average calculation with a different signed value from the others. Because the individual variation in the absolute duration of the MAE was large, we performed statistical tests after normalizing the data by computing a Z-score for each condition for each participant. The MAE duration difference between each condition and the grand average (over all conditions) was divided by the grand standard deviation (of all conditions). This removed the individual variations of absolute MAE duration and also the individual variations of effect size among different conditions. A one-way ANOVA showed that the main effect of the motion conditions was significant in both the static test (F(6, 28) = 13.29, p < 0.001), and the flicker test (F(6, 28) = 3.3, p < 0.05). A paired t-test with Holm’s correction showed that in the static test, the MAE durations for Rotation, Expansion/Contraction and Shear were significantly longer than that for the single patch condition (t(5) = 6.76, p < 0.05, t(5) = 4.76, p < 0.05 and t(5) = 4.75, p < 0.05)) while the MAE durations for other motion conditions were not significantly different from the single patch condition. In the flicker test, on the other hand, only one condition was statistically different from the single patch condition, the uniform motion condition, which showed a *shorter* rather than a longer MAE duration than a single patch (t(5) = 5.07, p < 0.05).

**Table 1:** Z-score and Hedges’ g of Experiment 1.

	Rotation	Expansion/contraction	Shear	Distortion	Random	Uniform	Single
**Static test**							
Average (Z-score)	0.67	1.44	0.46	− 0.52	− 0.58	− 0.76	− 0.71
SD	0.46	0.45	0.49	0.67	0.37	0.73	0.43
Hedges' g	2.19	3.47	1.82	0.24	0.24	− 0.05	−
**Flicker test**							
Average (Z-score)	− 0.20	− 0.30	0.20	0.38	0.66	− 1.23	0.48
SD	0.76	1.13	0.68	0.51	0.67	0.42	1.09
Hedges' g	− 0.52	− 0.50	− 0.22	− 0.09	0.14	− 1.47	–

### Discussion

The Rotation, Expansion/Contraction and Shear conditions produced significantly longer MAE durations than a single Gabor patch condition under the static test condition, suggesting that the output of the slow motion mechanisms shared the same mechanisms as the global motion systems that computed rotation, expansion/contraction, and also relative motion. The significantly shorter MAE for uniform motion than the single condition in the flicker test could also be attributed to the motion signal integration in space. One possible interpretation is lower sensitivity for stimulus with no or weak relative motion signal. Indeed, a previous study reported that participants’ sensitivity to relative motion is higher than motion without relative motion components (i.e., uniform motion)^4^. In the present study, the relative motion components in the Uniform condition are composed of patch motion relative to the fixation point in the same way as in the Single condition except that the Uniform condition had contribution from four patches drifting in the same direction. How the spatial integration of all contributing patches might have weakened participants’ sensitivity and reflected in shortened MAE duration cannot be answered by current study design. In addition, there is a difficulty to explain why shortened MAE was only observed in flicker test but not in the static test—the strength of the static MAE in the Uniform condition was similar to that in the Single condition for static test. The shorter MAE for uniform motion in the flicker test may simply be due to random fluctuation, which happened to cause a statistical difference from the Single condition. We leave an interpretation of the shorter MAE for uniform motion in the flicker test for future studies.

In Experiment 2, we focused on the Rotation and Expansion/Contraction conditions and tested whether the MAE duration was associated with the strength of global motion. While it has been suggested that there are separate populations of neurons sensitive to the two types of global motion^34^, the results of Experiment 1 suggest contribution of slow motion mechanism is similar for rotation and for expansion/contraction: for both types of global motion, static MAE duration was about 10 s on average and it was about 4 s on average in flicker MAE. We, therefore, expected similar results between the Rotation and Expansion/Contraction conditions in the following experiments.

## Experiment 2: adaptation strength manipulation

We manipulated the adaptation strength in Experiment 2 to examine the origin of the MAE observations obtained in Experiment 1. If the MAE increases with stronger global motion signals during adaptation, it supports the idea that we are indeed tapping the global motion processes. Otherwise, the MAE should be independent of the global motion adaption strength.

### Participants

Six male participants volunteered for this experiment. All the participants had normal or corrected-to-normal vision and were kept naïve about the purpose of the experiment except for one author. All of them participated in the rotation condition and four of them also participated in the expansion/contraction condition.

### Stimulus and procedure

**Figure 4:**
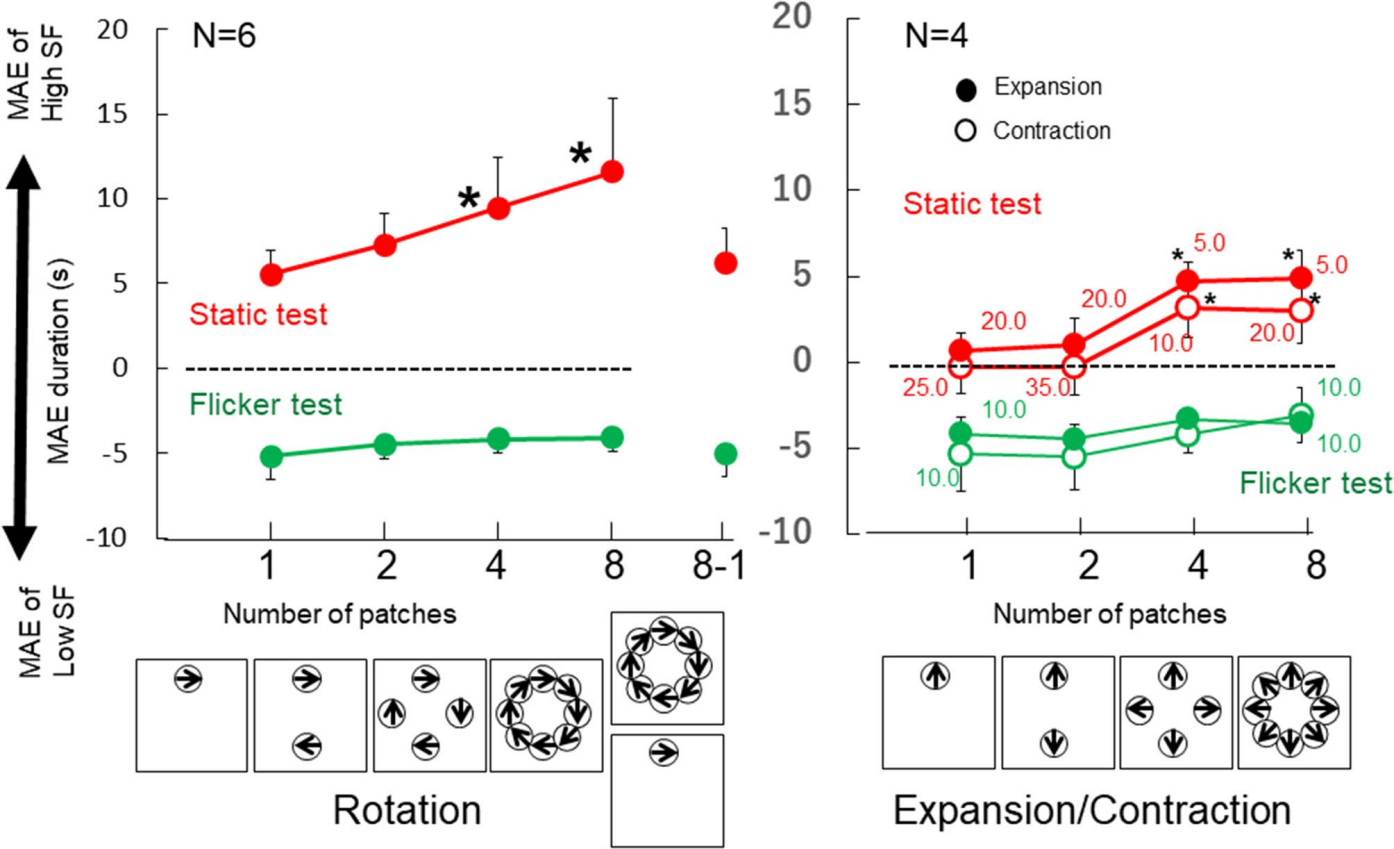
Results for Experiment 2. The MAE duration as a function of global motion strength. When the global motion strength was increased with more patches, the MAE obtained with the static test increased and the MAE direction was opposite to that of the high spatial frequency motion components. In contrast, the MAE obtained with the flicker test was in the opposite direction to that of the low spatial frequency motion components, and the magnitude did not vary with the global motion strength. Asterisks indicate that the MAE duration is significantly larger (p < 0.05) than that in the single condition. When there were responses opposite to the expected MAE direction in any condition, we provide the percentage of times that those occurred by a number near the data point for that condition. While this occurred frequently in the expansion/contraction conditions, it never occurred in any of the rotation conditions.

The adaptation stimuli were compound motion elements in a circular arrangement that moved to provide the sensation of expansion/contraction (Fig. 2A) or rotation (Fig. 2B). The stimuli and procedure were the same as those of Experiment 1 except for patch arrangements. The Gabor patches were arranged 4.5 degrees from the center (center to center distance) and the number of total patches in the display was varied to change the strength of the global motion: a single patch does not cause any rotation sensation and the degree of rotation sensation was expected to increase with the number of patches. The test stimulus was either a static or flicker version of the adaptation Gabor patches except for one condition, where eight patches were used in the adaptation and one patch was presented in the test (Fig. 4). This condition was included as a control to examine the effect of local and integrated global motion. The contrast of each grating was adjusted to be 30 times higher than the detection threshold measured with the method of adjustments. The average contrast for high SF grating was 31.7% (standard deviation: 5.2%), and 34.8% (standard deviation: 9.3%) for low SF grating. For all tests, MAE direction was judged by perception of global motion. Two keys were used to indicate clockwise/counter-clockwise (or expansion/contraction). For MAE during measurements, participants were asked to report the end of MAE as soon as any one element appeared to stop moving. The same criterion was used for all conditions across different patch numbers. This criterion was set differently from Exp. 1 because the number of patches is the main manipulation in Exp. 2 and 3 and we wanted to direct our participants’ attention to the whole pattern (instead of a single patch). With this criterion, if the MAE duration increases with adaptation patch number, we can be confident that we are tapping global motion processing.

### Results

The direction and duration of the MAE are shown in Fig. 4, and Table 2 shows MAE duration in Z-score and Hedges' g as the effect size of difference from the one patch condition. The results showed that the MAE direction was opposite to the high spatial frequency components in the static test (slow motion mechanisms dominated) and that of low spatial frequency components in the flicker test (fast motion mechanisms dominated) for all conditions except for single patch of contraction (the direction was defined by the high SF grating motion during adaptation). They confirmed the isolation of the slow and fast motion mechanisms as in the previous experiment.

**Table 2:** Z-score and Hedges' g of Experiment 2.

	1	2	4	8	8-1
**Rotation**					
Static test					
Average (Z-score)	− 0.85	− 0.34	0.60	1.31	− 0.72
SD	0.08	0.17	0.15	0.12	0.21
Hedges' g	0.00	1.12	3.54	6.03	0.24
Flicker test					
Average (Z-score)	0.36	− 0.30	− 0.19	0.31	− 0.19
SD	0.33	0.42	0.31	0.41	0.42
Hedges' g	0.00	− 0.50	− 0.49	− 0.04	− 0.42

	1	2	4	8	
**Expansion**					
Static					
Average	− 0.94	− 0.74	0.84	0.83	
SD	0.11	0.12	0.14	0.3	
Hedges' g	0.00	0.61	5.03	5.16	
Flicker test					
Average	0.77	− 0.34	− 0.22	− 0.21	
SD	0.55	0.27	0.24	0.55	
Hedges' g	0.00	− 0.92	− 0.83	− 0.63	
**Contraction**					
Static					
Average	− 0.82	− 0.86	0.90	0.78	
SD	0.15	0.13	0.09	0.08	
Hedges' g	0.00	− 0.09	4.76	4.57	
Flicker test					
Average	− 0.04	− 0.39	0.02	0.40	
SD	0.66	0.34	0.52	0.25	
Hedges' g	0.00	− 0.23	0.04	0.31	

The MAE duration in the static test increased with the number of patches. The MAE duration under the eight-patch condition was more than double that under the single patch condition. In contrast, the MAE duration was approximately constant across the number of patches in the flicker test. These patterns applied to both the rotation and expansion/contraction conditions. A one-way ANOVA was applied to normalized MAE durations (Z score) under Rotation. The results showed that the MAE durations with different patch numbers varied significantly in the static test (F(4, 25) = 36.91, p < 0.001), but not in the flicker test (F(4, 25) = 0.67 p > 0.1). A paired t-test with Holm’s correction showed that in the static test, the MAE durations for 4 and 8 patches were significantly longer than that for a single patch (t(5) = 13.04, p < 0.001 and t(5) = 13.21, p < 0.001), while the MAE durations for 2 patches and the MAE for the 8–1 condition did not differ from that for the single patch condition (t(5) = 2.79, p = 0.08 and t(5) = 0.49, p > 0.1).

We applied a two-way ANOVA test (expansion/contraction x patch number) to the results. For static test, we discovered a significant main effect on patch number (F(3,9) = 18.9, p < 0.001), but not on motion type (F(1,3) = 6.6, p = 0.082) or the interaction between patch number and motion type (F(3,9) = 0.37, p = 0.77). For flicker test, there was no significant effects for patch number (F(3,9) = 0.72, p = 0.57), motion type (F(1,3) = 7.97, p = 0.067), or the interaction (F(3,9) = 0.36, p = 0.78 for). Because the expansion/contraction results were similar, we collapse the two conditions. The paired t-test with Holm’s correction for the static test showed that the MAE durations for 4 and 8 patches were significantly longer than that for a single patch (t(3) = 9.28, p = 0.053 and t(3) = 12.03, p = 0.0037 for expansion; and t(3) = 12.92, p = 0.002 and t(3) = 7.35, p = 0.015 for contraction) while the MAE duration for 2 patches was not (p > 0.1 for both expansion and contraction). The same test showed that there was no statistical significance in the flicker test for all patch numbers of both types of motion (ps > 0.1). These results indicate that there is clear effect of patch number on expansion and contraction as well as on rotation.

### Discussion

Experiment 2 examined the different effects of patch number for static and flicker MAEs. Longer MAEs with more patches can be attributed to the influence of the process that integrates motion signals from multiple patches, namely a global motion detector. Such patch-number dependence is exclusively found under a static MAE, indicating that a slow motion mechanism is sensitive to global motion while a fast motion mechanism is not. The results confirmed the important contribution of slow motion mechanism to global motion.

**Figure 5:**
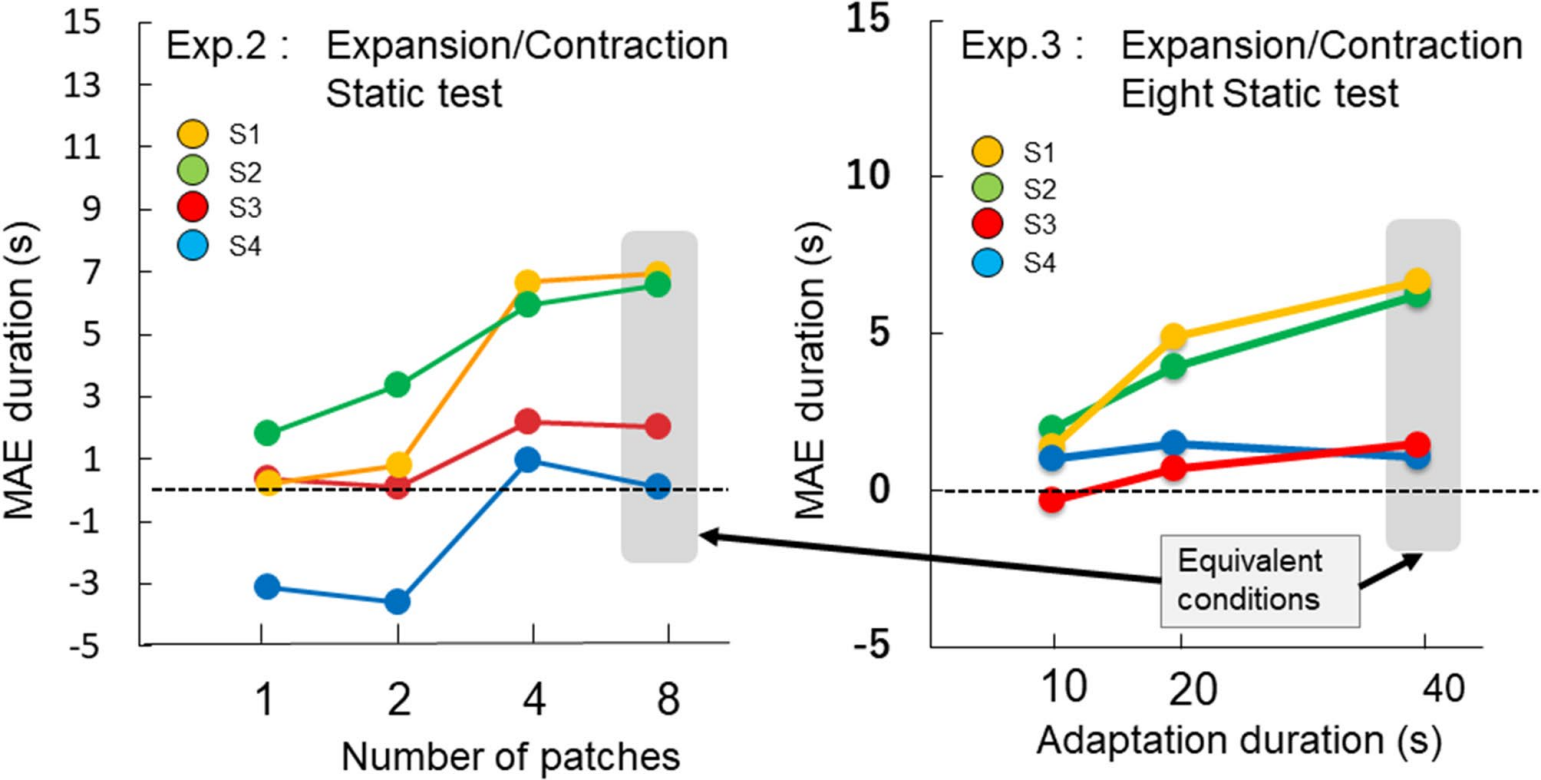
Results of individual participants of expansion/contraction in Experiment 2 (left) and in Experiment 3 (right). The 8-patch condition in Exp.2 and the 40 s condition in Exp. 3 are equivalent condition. The same four participants took part in the expansion/contraction conditions in both Exps 2 and 3.

There are results that do not follow our assumption of high SF dominance in static MAE for expansion/contraction motion conditions: negative static MAE was observed frequently in the single and two-patch conditions (more than 20% of all trials in some cases, see Fig. 4 right). This is because one participant's responses to static MAE were opposite to the low spatial frequency motion on average. The reported durations from the other participants were also shortened in the single and two-patch conditions (although their results followed our predicted direction), resulting in near-zero average (Fig. 5 left). This trend is within expectation in the present experiment. We set contrasts of low SF and high SF gratings to be equal in terms of detection threshold, using four-patch stimuli. As our current results suggested, slow motion mechanism is more sensitive with larger number of patches, but sensitivity of the fast motion mechanism appears to be constant across different patch numbers. The relative contribution from the fast motion mechanism (low SF grating motion) can be larger than that of the slow motion mechanism (high SF grating motion) in the single and two-patch conditions for some of participants. In such a case, static MAE direction will be in opposite of low SF grating motion. Therefore, static MAE direction is sometimes opposite to low SF motion direction, instead of opposite to high SF motion direction, particularly with small numbers of patches. These responses do not challenge the logic of our experiment, although this may be a limitation of our method to isolate two motion mechanism with static and flicker MAEs.

The MAE duration under the single patch condition after an eight-patch adaptation is similar to that under the single patch condition, where only one patch was used for adaptation. The global motion signal did not increase MAE duration, contrary to the case of eight patch test condition. That is, it is required for the effect of global motion to stimulate with global motion arrangement both during adaptation and test. This suggests that the increase of static MAE duration with patch number can be attributed to the process to analyze global motion, instead of the local motion detector which has influence through global motion process by feedback during adaptation. Based on these results, we assert that the outputs from the slow-motion mechanisms were the primary source for realizing global motion perception, at least under the present condition.

**Figure 6:**
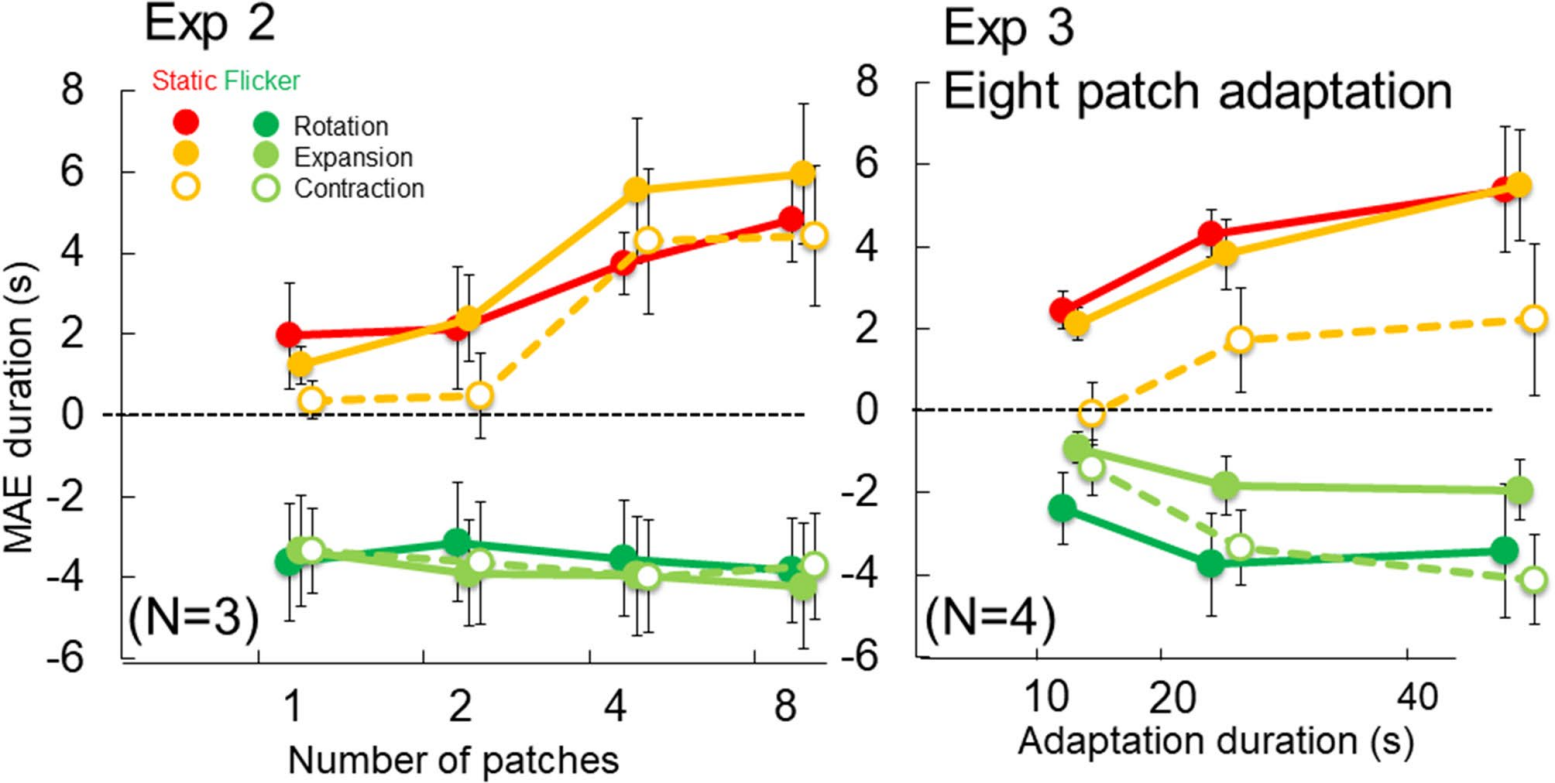
Comparison of rotation and expansion/contraction. The left panel shows results of three participants who took part in the rotation and expansion/contraction in Experiment 2 (effect of patch number), and the right panel shows results of four participants who took part in the rotation and expansion/contraction in Experiment 3 (effect of adaptation duration for eight patch adaptation).

Figure 4 shows that the static MAE for rotation is much longer than for expansion/contraction. Since the contrast was separately adjusted for each adapting global motion type based on detection thresholds, the MAE duration difference cannot be attributed to the motion strength difference during adaptation. Alternatively, that could be due to motion types (rotation vs expansion/contraction)^35–37^ or to individual differences. The individual variation of MAE duration was large in the present experiments, so we compared results from the three participants who took part in both extraction/contraction and rotation conditions in Experiment 2 (Fig. 6 left). We applied a repeated two-way ANOVA (motion type and patch number) separately for static and flicker MAE. For static MAE, we discovered statistically significant main effect of patch number (F(3,6) = 63.14, p < 0.001) but not motion type (F(2,4) = 2.03, p = 0.25). The results for flicker MAE showed no significant main effects of patch number (F(3,6) = 4.25, p < 0.063) and motion type (F(2,4) = 3.20, p = 0.15). No clear difference was found among different motion types after considering individual differences, so we speculate the visible MAE duration differences in Fig. 4 between rotation and the expansion/contraction is due to individual variation of participants’ sensitivities.

If the MAE size modulation caused by global motion strength in Experiment 2 reflected the motion detector sensitivity to global motion, the natural question to ask next relates to where the global motion integration occurred. This is important because it may help us elucidate the critical neural site that is the location of the slow and fast motion mechanisms. We addressed this question in the next experiment.

## Experiment 3: motion integration during adaptation and test stage

It is well known that global motion adaptation involves multiple stages^7,38^. The perceived global motion after adaptation to stimuli can be attributed to a sensitivity change in the global motion detector. Alternatively, it can be attributed to a sensitivity change in the local motion mechanisms, whose outputs are integrated to generate global motion perception. The two possibilities are not necessarily exclusive.

In Experiment 3, we tested these possibilities by reducing the strength of the global motion during the adaptation stage through dividing the original adaptation display into two sequential displays each containing half of the elements. When the sequential displays were viewed individually, weaker global motion was observed. In contrast, the summation of the two sequential displays provided the same amount of local motion energy at each location as the original global motion display.

### Participants

Six male participants participated in the rotation condition and four of them also participated in the expansion/contraction condition. All the participants had normal or corrected-to-normal vision and were kept naïve about the purpose of the experiment except for one who was an author.

### Stimulus and procedure

**Figure 7:**
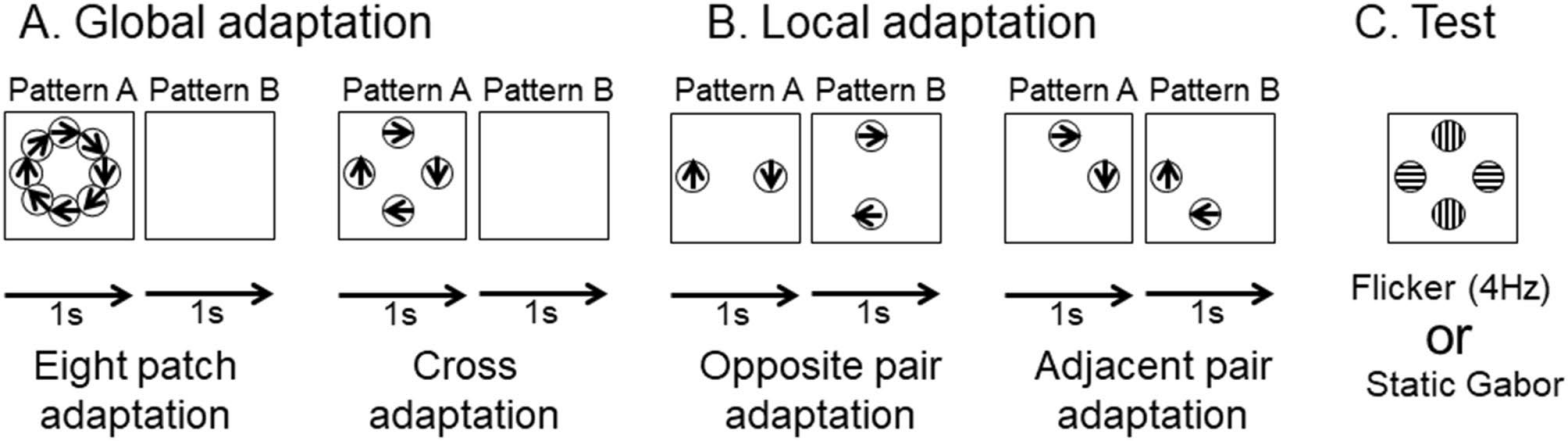
Stimuli of Experiment 3. (**A**) Global Motion Adaptation stimuli. Participants adapted to two alternating displays each consisting of eight or four Gabor patches forming a global rotational motion. Each display remained for 1 s, and the entire adaptation lasted for 10, 20, or 40 s. The presentation order of Patterns A and B were changed in half of sessions to minimize the difference of motion stimulus right before test presentation among conditions. (**B**) Local Motion Adaptation Stimuli. The alternating display was similar to the global motion condition except that each adapting display consisted of only two Gabor patches. The presentation order of Patterns A and B were changed in half of sessions as well. (**C**) The test stimulus was a display consisting of 4 Gabor patches, either static or flickered at 4 Hz. The participants used two keys assigned to opposite directions to report their perceived MAE when the MAE disappeared.

The stimuli were the same as those of Experiment 2 except for patch arrangements. We used four different adaptation conditions, in which two displays alternated at 1 Hz throughout the adaptation stage. In the global motion adaptation, eight or four patches were presented simultaneously (termed “Eight patch condition” and “Cross-condition” respectively, Fig. 7A) while under the local motion adaptation condition, two of the four patches were presented alternately (termed “Opposite-pair or OP” and “Adjacent-pair or AP” conditions, Fig. 7B). The test display was identical for all the adaptation conditions. It consisted of four Gabor patches located above, right, below and left of the fixation (Fig. 7C). According to our informal observation, global motion was perceived under two global adaptation conditions while it was not under the other two local adaptation conditions. The contrast of each grating was adjusted to be 30 times higher than the detection threshold measured with the method of adjustments. The average contrast for high SF grating was 32.3% (standard deviation: 4.6%), and 34.8% (standard deviation: 9.3%) for low SF grating.

After 10, 20 or 40 s adaptations, the MAE duration was measured in the static or the flicker (4 Hz) version of the four Gabor patches. The participants pressed one of the two response keys to indicate the perceived MAE direction (clockwise or counter-clockwise) as soon as any one element appeared to stop moving (i.e. the same as in Experiment 2). A third button for no MAE was also provided and when this was pressed, the MAE duration was recorded as 0.

### Results

**Figure 8:**
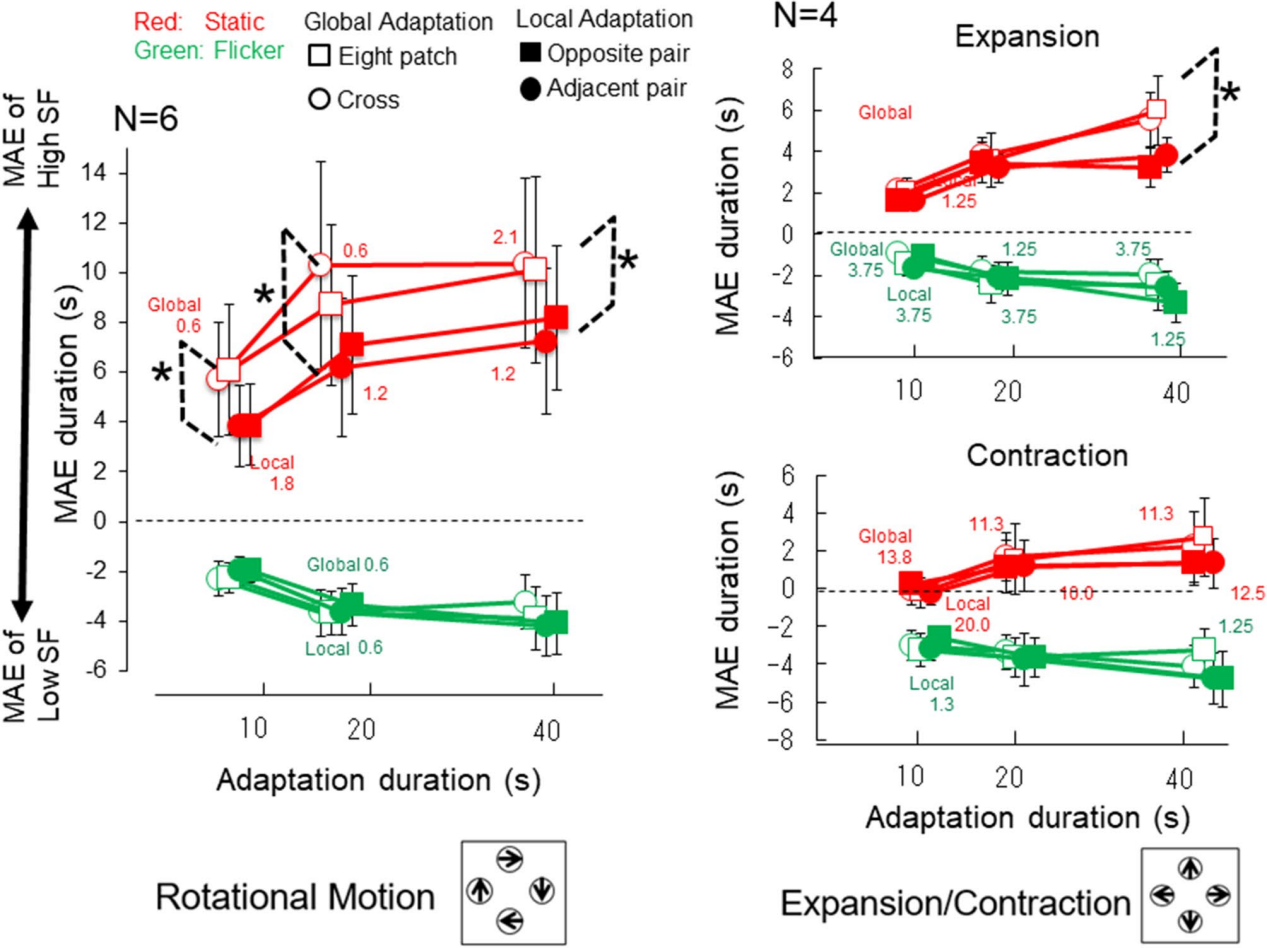
Results of global (open symbols) and local (filled symbols) adaptation motion in Experiment 3. The MAE duration as a function of adaptation duration. Both rotation (left) and expansion (right top)/contraction (right bottom) produced similar patterns. The MAEs obtained after employing the global and local adaptation conditions were significantly different with the static test, and the MAE direction was in the opposite direction to the high spatial frequency motion components. In contrast, the MAEs obtained with the flicker test, whose direction was opposite to that of the low spatial frequency motion components, were similar after global adaptation and local adaptation. Asterisks indicate statistical significance (p < 0.05). When there were responses opposite to the expected MAE direction in any condition, we provide the percentage of time that those occurred as a number near the data point for that condition.

The results are summarized in Fig. 8 (MAE duration in seconds), and Table 3 (Z-score and Hedges' g as the effect size of difference between the global and local motion conditions). For rotation, a two-way ANOVA for the normalized MAE duration (Z score) was conducted with factors of adapting condition (global or local adaptation) and adaptation duration (10, 20, 40 s) separately for the static test and the flicker test. In the static test, we found significant main effects of the adaptation duration (F(2,60) = 28.71, p < 0.001) and of the adapting condition (F(3,60) = 10.89, p < 0.001). No significant interaction between the two (p > 0.1) was reported. The result was similar for the expansion/contraction condition.

**Table 3:** Z-score and Hedges' g in Experiment 3.

	10 s adaptation	20 s adaptation	40 s adaptation
**Rotation**			
*Global*			
Static test			
Average (Z-score)	− 0.50	0.80	0.98
SD	0.43	0.60	0.75
Flicker test			
Average (Z-score)	− 0.75	0.43	0.22
SD	0.49	0.61	0.98
*Local*			
Static test			
Average (Z-score)	− 1.15	− 0.12	− 0.03
SD	0.32	0.41	0.73
Flicker test			
Average (Z-score)	− 1.03	0.18	0.94
SD	0.23	0.58	0.79
*Hedges' g: global versus local*			
Static	1.20	1.27	0.96
Flicker	0.52	0.30	− 0.57
**Expansion**			
*Global*			
Static test			
Average	2.08	3.70	5.75
SD	0.97	2.14	2.89
Flicker test			
Average (Z-score)	− 1.19	− 2.13	− 2.20
SD	0.86	1.65	1.88
*Local*			
Static test			
Average	1.60	3.32	3.52
SD	0.67	1.64	1.80
Flicker test			
Average	− 1.37	− 2.12	− 3.00
SD	1.16	1.70	1.90
*Hedges' g: global versus local*			
Static	0.41	0.14	0.65
Flicker	0.13	0.00	0.30
**Contraction**			
*Global*			
Static test			
Average	− 0.15	1.64	2.47
SD	1.53	3.16	3.93
Flicker test			
Average	− 3.13	− 3.50	− 3.69
SD	1.63	1.92	2.11
*Local*			
Static test			
Average	− 0.01	1.18	1.38
SD	1.15	2.71	2.54
Flicker test			
Average	− 2.90	− 3.71	− 4.79
SD	1.56	2.14	3.02
*Hedges' g: global versus local*			
Static	− 0.07	0.11	0.23
Flicker	− 0.10	0.08	0.30

Because the eight-patch and cross conditions were similar, we combined them into one global adaptation condition. Similarly, the OP and AP conditions had similar trends, so we combined them as the local adaptation condition. For static test, the MAE durations under the global adaptation condition were significantly longer than the local adaptations for all three exposure durations (t(5) = 3.90, p < 0.05 for 10 s adaptation; t(5) = 4.08, p < 0.01 for 20 s adaptation; and t(5) = 6.00, p < 0.01 for 40 s adaptation). The same statistical test performed under the flicker condition showed no significant effect for all adaptation durations (ps > 0.1 for 10 and 20 s and it is p = 0.09 with t(3) = 2.10).

For expansion/contraction, we applied a three-way ANOVA test (expansion/contraction x adaptation duration x adaptation type) separately for static and flicker MAE tests. For static test, the ANOVA showed significant main effects of motion types (i.e. expansion/contraction) (F(1,3) = 11.90, p = 0.041), adaptation duration (F(2,6) = 7.11, p = 0.026) and interaction between adaptation duration and adaptation type (F(6,16) = 3.27, p = 0.024). The effect of adaptation type was marginally significant (F(3,9) = 3.76, p = 0.053). For flicker test, the test showed significant main effects of expansion and contraction (F(1,3) = 14.62, p = 0.032) and adaptation duration (F(2,6) = 10.89, p = 0.010). No other effect was statistically significant (ps > 0.1). The comparison test between global and local adaptation conditions was performed separately for expansion and contraction. The results showed significant difference between the global and location adaptation conditions for 40 s adaptation of expansion (t(5) = 4.79, p = 0.017) while there was no statistical significance for other conditions.

These results indicate MAE was mainly from the global motion process during adaptation, not by integration of local MAE signals during the test phase. Because only static MAE showed a clear advantage in global adaptation condition, it is consistent with the idea that slow motion signals (assessed by static MAE) contribute to the global motion process more strongly than fast motion signals (assessed by flicker MAE). We observed noticeable differences across motion types. First, the strength of rotation motion appears to depend on patch arrangements more than expansion/contraction in the present conditions, suggested by the fact that the MAE durations between global and local motion adaptations is significantly different even for 10 s adaptation. Second, we found a stronger static MAE for expansion than contraction, possibly due to the stimulus contrasts because we did not measure contrast thresholds separately for expansion and contraction. In general, the present results confirmed that the slow motion signals contribute significantly more than fast motion signals to rotation and expansion. While there is a similar trend in contraction, it was not supported by the statistical test. We speculate that slow motion mechanism contributes to all of the three types of global motion similarly but with variable effect sizes.

### Discussion

Figure 8 shows that the static MAE for rotation is much longer than for expansion/contraction as in Fig. 4. We compared their results of eight patch condition for extraction, contraction and rotation as shown in Fig. 6 (right). We found that the differences among conditions are smaller if results of the same participants are compared. A two-way ANOVA (motion type and adaptation duration) for static test showed statistically significant main effect of motion type (F(2,6) = 5.87, p = 0.030) , and marginal main effect of adaptation duration ((2,6) = 4.55, p = 0.063). The same two-way ANOVA for flicker test showed no significant main effect (F(2,6) = 0.89, p = 0.46 for motion type and F(2,6) = 2.02, p = 0.21 for adaptation duration). Although it is likely that static MAE is shorter for contraction than for the other two, post multiple comparisons showed no statistical significance in difference among motion types. The analysis suggests that the large differences between rotation and the expansion and contraction seen in Fig. 8 is most likely due to different sensitivities among individual participants at least in part. The small difference between global and local motion adaptation for contraction could be related to its weak static MAE. Short MAE durations make reliable differences between conditions difficult to be confirmed by statistical tests.

The results of Exp. 3 supports the interpretation of the results from Exp. 2. There is one possible criticism from the interpretation of similar MAE durations with different patch numbers in Exp. 2 by null effect of flicker test. Without a direct measurement of absolute sensitivities, the null effect of flicker test in Exp. 2 could have an alternative explanation. The MAE enhancement by the fast motion mechanisms may be canceled out by the MAE enhancement caused by the slow motion mechanisms operating in the opposite direction. Figure 8, however, showed that both the static and flicker MAE durations increase with longer adaptation while only the static MAE showed a significant difference between the local and global conditions. There is no hint of cancelation between the two MAE components, which may hide the global motion effect on flicker MAE.

## General discussion

In this study, we investigated the spatiotemporal aspects of global motion with three experiments, and they all allowed us to draw the conclusion that a slow motion mechanism played a more important role in global motion processing than a fast motion mechanism. This observation applied to rotation, expansion/contraction, and shear motion but not to uniform motion (Experiment 1). When global motion adaptation strength was enhanced by increasing the number of local elements (Experiment 2) and by temporal simultaneity (Experiment 3), the MAE strength from the slow motion mechanism (revealed with a static test) was increased. Our most significant finding was that slow motion played a significant role in global motion processing, while we found no clear effect in fast motion of an increase in the number of local elements or from the temporal simultaneity of the elements.

Our major finding may appear inconsistent with other studies that suggested involvement of both the slow and fast motion mechanisms in global motion integration. However, the present results do not necessarily reject the contribution of a fast motion mechanism to global motion. Rather, they suggest that the contribution from a slow motion mechanism to global motion can be greater than that of a fast motion mechanism, at least under our experimental conditions. Perhaps, relative contributions of two motion mechanisms depend on stimulus conditions. A comparison of experimental conditions used in current and previous studies should assist an understanding of the differences between the roles of slow and fast motion mechanisms as regards global motion. Here, we discuss the differences between the experimental conditions of the present and previous experiments.

Our stimuli were overlapping Gabor patches, moving in opposite directions for adaptation. The method compares MAE strengths directly for the two types of motion mechanisms, and the two systems are easily isolated from each other by the MAE direction. Therefore, the effect of global motion can be investigated in isolation, using stimuli with different spatial frequency contents. Van der Smagt et al.^24^ reported a similar MAE study with transparent motion composed of two groups of dynamic random dots differing in speed (slow at 1.3 and 4 deg/s, and fast at 12 and 36 deg/s). When tested with a pattern containing both static (0 Hz) and dynamically-flickered (45 Hz) dots, the participants perceived a transparent MAE, which was believed to indicate that two motion sensor populations at selective speed ranges were tapped. Since the ability to see two surfaces of transparent motion requires global motion processing, their results suggest that both the fast and slow motion mechanisms contribute to global motion. One big difference between their and our studies is the spatial patterns used. We varied spatial frequency (holding temporal frequency constant) to manipulate speed differences in the two experimental conditions, while van der Smagt et al^24^ used random-dot patterns which have broad spatial frequency content. Although the speeds in these two studies were not very different (0.5 c/deg and 5 Hz in current study, 10 deg/s in study 24), the fast motion mechanism may be activated more from random dots in a large field. Spatial integration size is smaller for high spatial frequency stimuli than for low spatial frequency stimuli so that motion in a larger field stimulates the fast motion mechanism more^39,40^. Our stimuli covered about 25 deg by high contrast areas (±  σ where contrast was larger than 0.18 with the peak contrast of 0.3) of eight Gabor patches while the stimuli used by van der Smagt et al^24^ covered about 50 deg. It is possible that our stimulus condition was not appropriate for the fast motion mechanism in terms of stimulus size. If stimulus size was smaller than the integration size of a mechanism, its contribution might be underestimated. In addition, even when the size was the same, it is also possible that Gabor stimuli and random dot stimuli stimulated the two motion mechanisms differently if we consider neural selectivity to orientations. Random dot patterns stimulate with a variety of orientation components whereas Gabor patches do with one major orientation. Because the fast motion mechanism has a broader orientation tuning^41^, random-dot patterns are expected to elicit larger neural outputs sensitive to fast motion than Gabor patches. In contrast, it is possible that there is less difference in stimulation strength between these two stimulus types for neurons sensitive to slow motion and to a restricted orientation range.

Another series of studies employing a masking experiment also showed that motion mechanisms tuned to slow and fast speeds contribute to global motion. Edwards, Badcock, and Smith^30^ measured the participants’ minimum motion coherence threshold for direction identification while coherently-moving (signal) dots were masked by noise dots in various speed ranges. Their results showed that there were at least two independent speed-tuning global motion mechanisms; one sensitive at speeds below 3.6 deg/s and the other sensitive at higher speeds (above 10.8 deg/s). Random-dot patterns were used and the stimuli covered area larger than 200 deg^2^. In physiological studies of monkeys, random-dot patterns filling a visual field larger than 10 deg in diameter, which covers visual field of more than 75 deg^2^, were usually used to demonstrate faster speed tuning of global motion^42^. It is highly possible that the large field random-dot patterns stimulate the fast motion mechanism, which integrates signals from large areas independently of stimulus orientation, more than those with our stimuli (i.e. eight or four Gabor patches in the present experiment).

We found that a motion mechanism sensitive to high spatial frequencies contributes to global motion. This higher-spatial-frequency tuning is a unique feature of the global motion process discovered here. It was identified possibly because of our choice of smaller and higher spatial frequency stimuli than other studies. Previous motion integration studies employed large field or spatially broad band stimuli often show broadband^43^ or lowpass spatial frequency tunings^44,45^. The effect of global motion for small stimuli may be processed by the slow motion mechanism and that for large visual field be processed by the fast motion mechanism.

Past studies have suggested that the visual system relies on two motion mechanisms to master different global motion functions. Andersen and Braunstein^46^ proposed a two-stage model of self-motion perception, which includes a primitive mode involving a larger area at the periphery and a higher visual mode sensitive to more complex optical flow patterns mediated by central vision. Palmisano and Gilliam^47^ also reported that vection (i.e. self-motion perception) induced in the central vision was more compelling for high-spatial-frequency optic flow patterns, while peripherally mediated vection was more compelling for low-spatial-frequency flow patterns. Since global motion processing is critical for the visual perception of self-motion, these studies support the contribution of two motion mechanisms to global motion, perhaps for different purposes: one uses detailed information from the central visual field and the other uses coarse information from the periphery. The slow motion mechanism sensitive to high spatial frequencies can be considered to contribute to global motion at a central visual field with analysis of detail information.

Several recent studies have also offered insights into possible neural substrates responsible for multiple global motion processes while the relationship with functions is not clear. Global motion is believed to be processed in middle temporal area (MT) and medial superior temporal area (MST). Monkey neuronal responses in MT and MST were linearly proportional to global motion coherence^48,49^. In the human visual system, the bold signals in the motion area V5/MT + increased with global coherence^50,51^, while V1 tends to exhibit a negative response to increasing coherence global motion^52^. The earliest visual stage that responds to complex motion (e.g. circular, radial, and spiral motion) is likely the medial superior temporal region (MSTd) in the dorsal region of the macaque monkey^8,42^ and in the human V5/MT + complex^53,54^. While there is a consensus view that global motion perception must involve pooling information from neural sites earlier than MST, the way in which the computation operates remains to be determined. MST should receive signals through the cortical visual pathway via V1. However, there is a direct subcortical connection via the pulvinar or LGN^55^ and a callosal connection with the contralateral hemisphere^56^. Clinical studies from patients with damaged V1 showed that their V5/MT responses continued to be shaped with global motion coherence, possibly via inputs from intact residual pathways such as subcortical inputs^57^.

There are at least three possible multi-channel motion pathways in the visual system related to global motion perception. Firstly, in monkey MT, two different populations of neurons were identified: one sensitive to relative motion at adjacent areas while the other to the same motion in the large area^58^. They may correspond to the slow and fast motion mechanisms which could imply that global motion is processed within MT differently based on spatiotemporal differences in stimulation. Secondly, differences in spatiotemporal sensitivity of the fast and slow motion mechanisms are similar to those between the magno- and parvo-pathways (sensitivity to high temporal and low spatial frequencies vs sensitivity to low temporal and high spatial frequencies). Shioiri and Matsumiya^23^ discussed this issue and summarized that there is no concrete evidence to support this possibility. Thirdly, motion signals in cortical and subcortical pathways both convey signal used for perceiving global motion in different ways. All of these possibilities provide reasons to consider more than one pathway for global motion while further investigation is required in order to identify the physiological counterparts of the slow and fast global motion mechanisms.

Here we use the flicker MAE to probe the fast motion mechanism, and flicker MAE has been used to investigate motion analysis at higher stages, which are influenced by attention and/or are sensitive to second order motion^59–62^. When the first and second order motion components in a stimulus move in opposite directions, MAE is observed in the direction opposite to the first order motion in a static test while MAE in the direction opposite to the second order motion in a flicker test^62^. The effect of attention for current MAE paradigm was examined by Shioiri and Matsumiya^23^ by measuring static and flicker MAEs with and without a central attentional task, where the observer detected digits in a rapidly presented letter-and-digit sequence. When attention was removed from the adapting motion stimuli, both static and flicker MAEs reduced in similar amount. In other words, the distinct difference in temporal frequency tuning between static and flicker MAEs persisted (see Fig. 9 in Shioiri and Matsumiya), implying that attention is not a factor to differentiate the temporal frequency tunings between the two motion mechanisms. This finding leads us to assume that our flicker MAE in the present study corresponds to low level motion systems. On the other hand, the relationship with the second-order motion is not clear. The second-order motion mechanisms have been identified as independent processes from attention-based motion mechanisms^63,64^. It is theoretically possible that the different static and flicker MAE directions are due to the different contributions of the first-order and second-order motion mechanisms. Luminance gratings can stimulate the second-order, as well as the first-order and third-order motion systems^65–70^. If we assume that second-order motion mechanism is responsible to flicker MAE, we can still explain the results with replacing the fast motion mechanism by the second-order motion system. The story is the same as the dichotomy of slow/fast motion mechanisms, if we assume that the second-order motion mechanism has sensitivity to lower spatial and higher temporal frequencies than the first-order mechanism. However, this is not consistent with the temporal frequency tuning for the second-order motion mechanism, which is known to be lower than fast-order motion mechanism^71^. Furthermore, removal of luminance and other high-level modulations from the stimuli is required to measure the sensitivities of the second-order motion system in isolation. There is no reason to assume that the second-order motion mechanism is responsible to flicker MAE in our experiments because our stimuli were not designed to probe the second-order motion alone in current experiments.

Before offering our conclusion, we summarize the features of the experimental paradigm that we used here. Past studies on global motion have consistently suggested at least two speed-tuning mechanisms, but none of them described the spatiotemporal characteristics of these two mechanisms that render global motion perception^30,72–74^; possibly due to the fact that the stimuli commonly used in previous studies were random-dot motion sequences while motion integration is investigated with Gaussian or Gabor patches occasionally^7,44^. However, dots contain of the entire spatial frequency range and thus made it impossible to describe the spatial limits of these two speed-tuning systems. Our paradigm employing drifting Gabor patches as adapters had the following advantages. First, our multiple-aperture display requires spatial integration with large distances as each component is required to form a spatially-distributed contour. This avoids the short-range processing possible with a random-dot display where participants may be able to detect the global motion vector from a small cluster of dots. Secondly, each component is a Gabor patch separately defined by its orientation, spatial frequency, and phase. This enabled us to explore these dimensions separately. Finally, our compound Gabor is unique because it contains two superimposed gratings with different spatial frequencies (one higher than the other) and drift directions (180 deg difference). These two components can be used to tag and segregate separate mechanisms, which is impossible in displays containing one motion component. With our nicely designed set of both static and flicker tests, we were able to isolate each motion component and measure its MAE magnitude. This enabled us to describe the spatial limitation of fast and slow motion mechanisms that previous studies were unable to achieve, and made our stimuli and paradigm very favorable choices for future studies of this kind.

To conclude, we revealed a slow motion mechanism with sensitivity to high spatial frequencies that integrates motion signals over space. Similar spatial integration was not seen for the fast motion mechanism under the conditions used in the present experiment. This indicates that slow motion signals with detailed spatial information can be more important than fast motion signals under certain conditions such as for encoding object surfaces (structure from motion), depth from relative motion (motion parallax), and motion in depth through interocular velocity differences.

## Supplementary Information

Supplementary Information 1.

Supplementary Information 2.

Supplementary Information 3.

Supplementary Information 4.

Supplementary Information 5.

Supplementary Information 6.

Supplementary Information 7.
